# Decline in severe diarrhea hospitalizations after the introduction of rotavirus vaccination in Ghana: a prevalence study

**DOI:** 10.1186/1471-2334-14-431

**Published:** 2014-08-06

**Authors:** Christabel C Enweronu-Laryea, Isaac Boamah, Eric Sifah, Stanley K Diamenu, George Armah

**Affiliations:** Department of Child Health, University of Ghana Medical School, P O Box 4236, Accra, Ghana; Department of Microbiology, University of Ghana Medical School, Accra, Ghana; Princess Marie Louis Children’s Hospital, Accra, Ghana; World Health Organization, P O Box MB 142, Accra, Ghana; Noguchi Memorial Institute of Medical Research, University of Ghana, Accra, Ghana

**Keywords:** Diarrhea, Children, Rotavirus, Hospitalization, Vaccination, Ghana

## Abstract

**Background:**

Almost all diarrhea deaths in young children occur in developing countries. Immunization against rotavirus, the leading cause of childhood severe dehydrating acute diarrhea may reduce the burden of severe diarrhea in developing countries. Ghana introduced rotavirus and pneumococcal vaccination in the national expanded program on immunization in May 2012.

**Methods:**

Review of all-cause diarrheal hospitalization data for children aged 59 months and younger at 2 pediatric referral hospitals in southern Ghana from 2008 to 2014. The proportion of acute diarrhea (defined as 3 or more watery, non-bloody stools within 24 hours that has lasted for less than 7 days) cases caused by rotavirus was determined. Temporal trend and age group distribution of all-cause diarrhea and rotavirus gastroenteritis before and after introduction of the new vaccines were compared.

**Results:**

Of the 5847 children hospitalized with all-cause diarrhea during the 74 months (January 2008 – February 2014), 3963 (67.8%) children were recruited for rotavirus surveillance and stool specimens were tested for rotavirus in 3160/3963 (79.7%). Median monthly hospitalization for all-cause diarrhea reduced from 84 [interquartile range (IQR) 62 – 105] during the 52 months pre-vaccination introduction to 46 (IQR 42 - 57) in the 22 months after implementation of vaccination. Significant decline in all-cause diarrhea hospitalization occurred in children aged 0 - 11 months: 56.3% (2711/4817) vs. 47.2% 486/1030 [*p* = 0.0001, 95% confidence interval (CI) 0.77 – 0.88] and there was significant reduction of rotavirus gastroenteritis hospitalization: 49.7% (1246/2505) vs. 27.8% (182/655) [*p* = 0.0001, 95% CI 0.32 - 0.47] before and after vaccine introduction respectively.

**Conclusions:**

Implementation of rotavirus vaccination program may have resulted in significant reduction of severe diarrhea hospitalization even though this observational study could not exclude the effect of other confounding factors. Continued surveillance is recommended to monitor the progress of this program.

**Electronic supplementary material:**

The online version of this article (doi:10.1186/1471-2334-14-431) contains supplementary material, which is available to authorized users.

## Background

Globally, diarrheal diseases are the second leading cause of death among children under the age of 5 years [1]. Even though the disease burden from diarrhea has declined compared to the previous decade it remains a major cause of morbidity in developed countries while about 98% of diarrheal deaths continue to occur in low income countries [2, 3]. Diarrhea causes an estimated 25% of deaths in African children under the age of 5 years [2]. Acute gastroenteritis (AGE) is the most common presentation of diarrheal diseases. Rotavirus is the leading cause of severe dehydrating AGE in young children such that virtually all children would have experienced at least one episode of rotavirus infection before the age of 5 years [4]. Rotavirus is the commonest cause of AGE in young ghanaian children all year round [5–7]. The rotavirus peak infection season in Ghana occurs during the dry cool months of December – February but similar peaks have been observed during the cool wet months of May – July in the southern parts of the country.

Rotavirus infection is a preventable cause of childhood deaths; yet it accounted for approximately one-third of the 1.34 million diarrhea deaths and 9 million hospitalizations worldwide among children aged 59 months and younger in 2004 [8]. More recent global estimates in 2011 reveal that rotavirus caused 197,000 of the 700,000 diarrhea deaths [9]. In 2009, the World Health Organization (WHO) recommended that all countries introduce rotavirus vaccination into national expanded program for immunization to control severe rotavirus disease [10]. The WHO also recommended that countries conduct local surveillance studies prior to introduction of new vaccines [11]. The local WHO-sponsored surveillance study for rotavirus gastroenteritis became well established in Accra, Ghana in 2008. This report presents the trend in the burden of severe diarrhea in two referral pediatric hospitals before and after the introduction of the single-strain human rotavirus vaccine Rotarix® (GlaxoSmithKline Biologicals) in the expanded program on immunization in Ghana in May 2012.

## Methods

### Study site

The study was done at two pediatric referral hospitals in Accra Metropolis, Ghana’s capital city from January 2008 to February 2014. The population of Accra is estimated to be over 4 million and the 2 hospitals namely Children’s hospital and Korle Bu Teaching hospital are the major pediatric referral hospitals for children in Accra and surrounding regions of southern Ghana. Together, both hospitals have a capacity of over 250 pediatric beds and provide care for about 105,000 outpatients and 10,000 in-patients annually. Both hospitals usually document data on all children hospitalized overnight and/or for at least 24 hours in an admissions and discharges register.

### Study population

Data from all children aged 0 - 59 months and hospitalized with the diagnosis of diarrhea during the surveillance period were included in this analysis. However, only children with a primary diagnosis of AGE whose parents gave informed consent for the study met the inclusion criteria for the rotavirus surveillance study and stool specimen testing. Definition of AGE for the surveillance was 3 or more watery, non-bloody stools within a 24 hour period and of less than 7 days duration. The proposal for the surveillance study was submitted to the Ethical and Protocol Review Committee of University of Ghana Medical School in 2006. The study was considered to be of negligible risk to participants (field workers did not have any contact with the children and the inconvenience to parents involved providing clinical and demographic information that was not available in the child’s hospital folder and scooping the stool from their child into the specimen bottle provided) and exempted from full ethical review. Participation in the study was voluntary and no incentive was given to encourage participation.

### Data collection and laboratory analysis

Data on all diarrhea hospitalizations aged 0 - 59 months were collected from patients’ folders and the admissions and discharges register of the participating hospitals. Sampling strategy for the surveillance study consisted of daily patient enrollment from 8.00 am to 5.00 pm (except weekends and holidays). A standard questionnaire was used to collect clinical data (episodes of diarrhea, vomiting, fever, clinical signs, treatment given, outcome) from the parents and hospital folders of children enrolled in the rotavirus surveillance study. Microbiological data on the stools of children who were not enrolled in the rotavirus surveillance study was not collected. Stool specimens from children enrolled in the surveillance study were collected in labeled screw-top containers no later than 7 days after the onset of the illness and within 48 hours of hospitalization. Specimens were stored at 4°C until testing by enzyme immunoassay (EIA) using WHO approved kits (IDEIA™, DAKO Diagnostics, United Kingdom) for detection of rotavirus antigen. Rotavirus-positive specimens were sent to the West African Rotavirus Reference Laboratory at Noguchi Memorial Institute of Medical Research for determination of rotavirus genotypes. Quality control was ensured by re-testing 10% of EIA-negative stool specimens at the reference laboratory.

### Data analysis

All data were entered into Epi Info 3.5.1 database. We compared the monthly and age group distribution of all-cause diarrhea and rotavirus AGE before and after the implementation of rotavirus vaccination. Categorical variables were compared using the Pearson’s Chi squared test. Quantitative variables were compared using student’s *t*-test and one way analysis of variance. Probability value less than 0.05 was considered significant. All data were analyzed with SPSS version 16.

## Results and discussion

During the 74 months (January 2008 – February 2014) of the study there were 5847 diarrhea hospitalizations, 5537 (94.7%) were aged 0 – 35 months, 3963/5847 (67.8%) were recruited for rotavirus surveillance and 3160/3963 (79.7%) children had stool specimens tested for rotavirus. The median duration of hospitalization for diarrhea was 3 days with an interquartile range (IQR) of 2 – 5 days, and only 1.5% of all cases were hospitalized for more than 14 days.

### Temporal trend

**Figure 1:**
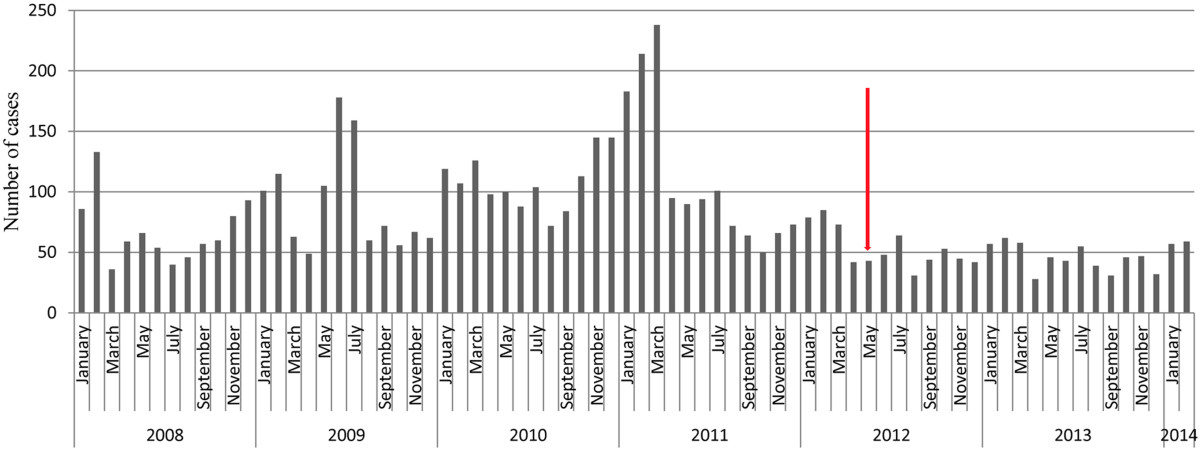
**Diarrhea hospitalizations among children aged 0-59 months at two referral hospitals in southern Ghana.** Arrow indicates the implementation of rotavirus immunization in May 2012.

**Figure 2:**
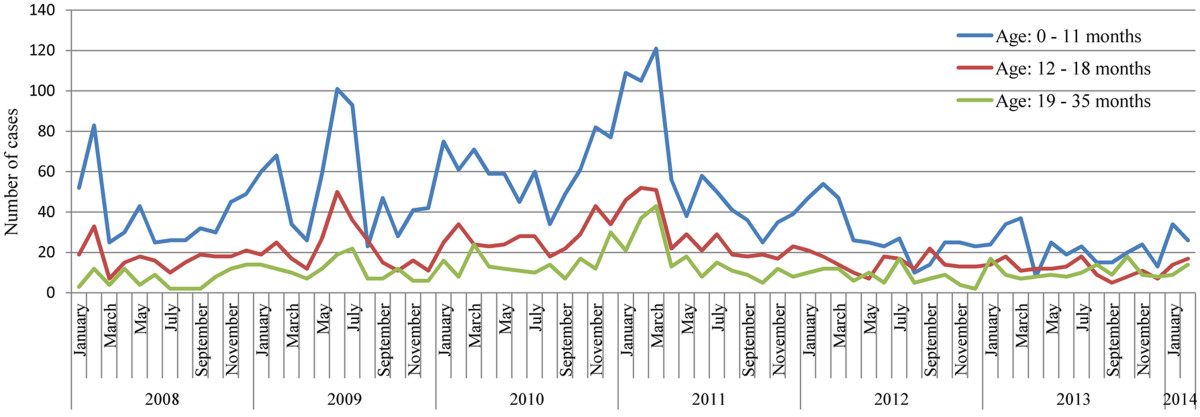
**Temporal trend of all-cause diarrhea hospitalization among different age groups of Ghanaian children.**

The total number of children hospitalized with diarrhea during the 52 months (Jan 2008 – April 2012) prior to introduction of rotavirus vaccination was 4817 and the median monthly hospitalization for the period was 84 (IQR 62 – 105) children. Of the 1030 children hospitalized in the ensuing 22 months (May 2012 – February 2014), the median monthly hospitalization was 46 (IQR 42 - 57). Diarrheal hospitalizations peaked during December – February every year (and also May – July in 2009) in the period prior to the introduction of rotavirus vaccination but these seasonal peaks were significantly blunted after the introduction of vaccination in 2012 (Figure 1). The yearly hospitalization for all-cause diarrhea (Table 1) showed a 51.6% and 16.2% decline from 2011 to 2012 and 2012 to 2013 respectively. The yearly prevalence of rotavirus AGE declined from an average of 50% during the pre-vaccine introduction years to 38% and 32% in 2012 and 2013 respectively.The most likely explanation for reductions in all-cause diarrhea hospitalization and blunting of seasonal peaks is the introduction of rotavirus vaccination as demonstrated in Figure 2. The burden of diarrheal hospitalizations was mostly among children aged 0 – 11 months, the vaccinated group. This age group was most affected during the peak seasons of diarrhea and they accounted for 56.3% (2711/4817) vs. 47.2% 486/1030 of all diarrhea hospitalizations before and after rotavirus vaccination introduction respectively.

**Table 1:** **Diarrhea hospitalizations and rotavirus surveillance data of children aged 0-59 months in southern Ghana**

Year of study	Diarrhea hospitalizations	Cases stool specimen tested (% diarrhea hospitalizations)	Cases with rotavirus positive stools	Annual prevalence of rotavirus gastroenteritis (%)
2008	810	379 (47)	190	50
2009	1087	443 (41)	233	53
2010	1301	662 (51)	284	43
2011	1340	805 (60)	427	53
2012	649	448 (69)	169	38
2013	544	358 (66)	113	32
*2014	116	65 (56)	12	18
**Total**	**5847**	**3160 (54)**	**1428**	

*Data for January and February 2014 only.

There was significant decline in the proportion of diarrheal hospitalizations occurring in children aged 0 - 11 months in the first year (transition year, May 2012 – April 2013) of vaccination with a relative increase in the proportion of hospitalizations among those aged 12 – 35 months (Table 2). Comparing the transition year with the second year of vaccination (May 2013 – February 2014), the proportion of 12 – 18 months age group hospitalized with diarrhea reduced from 30.3% (174/575) to 25.1% (114/455) [p = 0.064, 95% confidence interval (CI) 0.99 – 1.48] while the 19 – 35 months age group (the unvaccinated group) increased from 17.4% (100/575) to 23.7% 108/455) [p = 0.011, 95% CI 0.57 – 0.93]. There was no significant difference in hospitalization for children aged 0 – 11 months and 36 – 59 months in the second year. Similar observations have been reported by other workers [12]. These observations in the temporal trend of severe diarrhea hospitalization suggest a strong link to the implementation of rotavirus vaccination. However, this observational study cannot infer direct causal association between rotavirus vaccination and the prevalence of all-cause diarrhea.

**Table 2:** **Changes in diarrhea-related hospitalizations: comparing baseline and first year of rotavirus immunization in Ghana**

Age group (months)	Number of diarrhea hospitalizations		Proportion of diarrhea hospitalizations (%)		Relative change in hospitalization (95% CI)	***p***value
	Baseline (2008 - April 2012)	Transition year (May 2012 - April 2013)	Baseline (n = 4817)	Transition year (n = 575)	%	
0 - 5	1000	98	20.8	17.0	3.8 (1.01 - 1.47)	0.036
6 - 11	1711	174	35.5	30.3	5.2 (1.03 - 1.34)	0.012
12 - 18	1212	174	25.2	30.3	5.1 (0.73 - 0.95)	0.008
19 - 35	632	100	13.1	17.4	4.3 (0.62 - 0.75)	0.004
36 - 59	262	29	5.4	5.0	0.4 (0.74 - 1.57)	0.69

### Rotavirus gastroenteritis

Severe diarrhea requiring hospitalization occurred most commonly in children aged 0 - 18 months. This age group accounted for 81.4% (3923/4817) and 75.1% (774/1030) of all-cause diarrheal hospitalizations before and after the implementation of rotavirus vaccination respectively. Stools specimens of 3160/3963 children were tested for rotavirus, of these 2505 were tested in the 52 months before implementation of vaccination while 655 were tested in the 22 months of rotavirus vaccination. The prevalence of rotavirus gastroenteritis in children hospitalized with diarrhea decreased significantly: 49.7% (1246/2505) vs. 27.8% (182/655) [*p* = 0.0001, 95% CI 0.32 -0.47] as shown in Table 1. The proportion of children aged 0 – 11 months and hospitalized with rotavirus gastroenteritis was also significantly reduced in the transition year (May 2012 – April 2013) compared to the pre-vaccination period: 45.7% (43/94) vs. 72.6% (904/1246) [p = 0.014, 95% CI 1.04 – 1.72] respectively. The nation-wide coverage of rotavirus vaccination ranged from 42% - 56% in 2012 and 73% - 93% in 2013.

**Figure 3:**
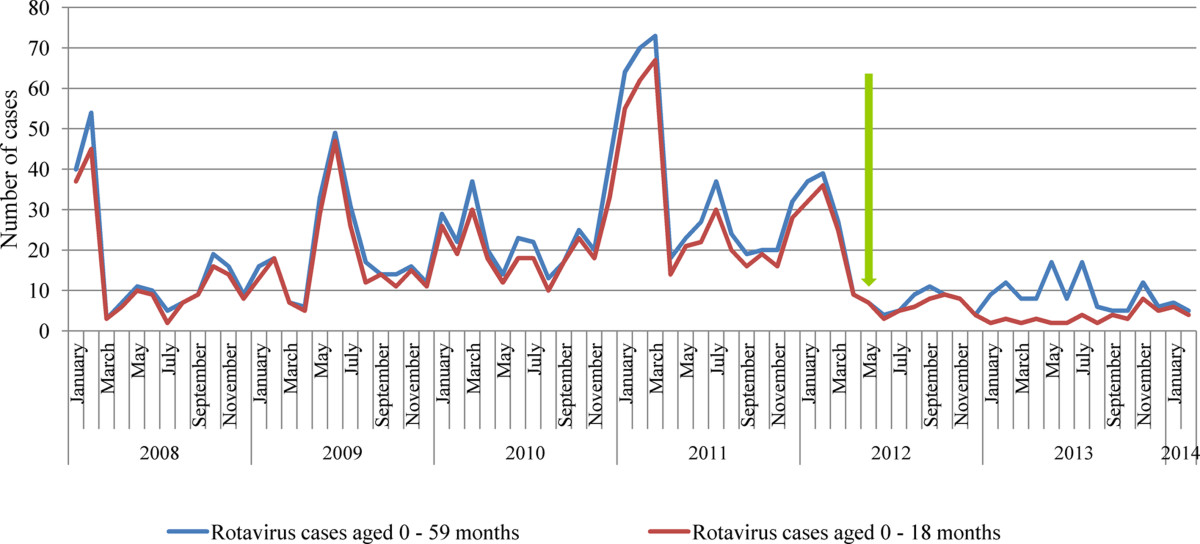
**Trend in rotavirus gastroenteritis hospitalizations among Ghanaian children aged 0-59 months: 2008 – 2014.** Arrow indicates the implementation of rotavirus immunization in May 2012.

Figure 3 shows the temporal trend of rotavirus gastroenteritis hospitalization. Children aged 0 – 18 months accounted for 87.3% (1088/1246), 63.8% (60/94) and 45.4% (40/88) of rotavirus diarrhea hospitalizations in the pre-vaccination period, transition year and the second year of vaccination respectively. Similar observations have been reported from other low and middle income countries [13–15]. Univariate analysis of variance showed a significant difference in the yearly trend of rotavirus hospitalization among children aged 18 months and younger from 2008 to 2013. However, further analysis with LSD (Least Square Difference) test showed that there was no difference between 2008 and 2012 (*p* = 0.812) but there was a significant difference between 2008 and 2013 (*p* = 0.036). The similarity between 2008 and 2012 could be explained by the fact that the vaccine introduction in May 2012 occurred after the 2012 peak diarrhea season in January and February. Linking these observations with the nationwide coverage of rotavirus vaccination suggests that the intervention has been effective in reducing severe diarrhea in the local population.

### Limitations

Severe diarrhea is a major cause of death among children in low income countries due to several factors including poor access to quality health care and delays in referral to a health facility. Prevention of severe diarrhea is particularly important in sub-Saharan African countries including Ghana because it is a leading cause of under 5 childhood deaths and imposes significant financial burden on impoverished families [16, 17]. The main objective for introducing rotavirus vaccination is to reduce diarrhea-related childhood deaths. This report has not addressed diarrhea deaths because even though we observed fewer deaths after vaccine introduction more data from a longer observation and wider population is required to assess the effect of rotavirus vaccination on childhood deaths. Other workers estimate that rotavirus vaccine has the potential to substantially reduce rotavirus-associated deaths in rural settings in Ghana [18].

Other possible limitations to this study include the inherent limitations of ecological studies as we cannot determine the risk of diarrhea of any cause for an individual child based on observations presented in this work. The relatively low number of stools (54% of all diarrhea hospitalizations) tested for rotavirus may have underestimated the real impact of vaccination. The blunted seasonal peaks observed after the introduction of rotavirus vaccination could have resulted from inherent changes in rotavirus seasonality or other confounding factors. However, we deem this most unlikely. First, data from pre-vaccination years makes this explanation improbable even though longer post-vaccination period may be required to confirm this observation. Secondly, there were no changes in hospital diagnostic coding for diarrhea documentation in the admissions and discharges registers of both hospitals during the 74 months of the study and we could not determine other changes in clinical practice to explain these findings.

A confounding factor of interest is the contribution of other health interventions. Ghana introduced the pneumococcal and rotavirus vaccines simultaneously in May 2012. Pneumonia and diarrhea are two infectious diseases that cause most morbidity and mortality in children aged 36 months and younger. The co-occurrence of both conditions in the same child (co-morbidity) has been shown to occur more frequently than can be explained by chance [19–21]. While the introduction of the pneumococcal vaccine may have impacted the decline in diarrheal hospitalization, existing data suggest that the risk of co-morbidity with pneumonia occurred in children with persistent diarrhea especially those with diarrhea that had lasted for 20 days or more [22]. Most children in this study did not have persistent diarrhea.

## Conclusions

The introduction of the single strain rotavirus vaccine Rotarix® was associated with substantial decline in hospitalization for all-cause diarrhea among children aged 59 months and younger in southern Ghana. All-cause diarrhea is not a precise measure of the effect of rotavirus vaccination on diarrhea hospitalization. However, in low-resource countries like Ghana stool testing for etiologic diagnosis of diarrhea is not routinely done in practice. In such circumstances, all-cause diarrhea provides the most valuable and practical measure of vaccine impact for public health and policy decision making. This work provides evidence of substantial reduction in diarrhea related hospitalizations following the introduction of rotavirus vaccination in southern Ghana. It also provides good evidence for health administrators and policy makers in Ghana to ensure good coverage for rotavirus vaccination and for other African countries who have not introduced rotavirus vaccination to review their policy.

## Authors’ original submitted files for images

Below are the links to the authors’ original submitted files for images. Authors’ original file for figure 1 Authors’ original file for figure 2 Authors’ original file for figure 3
