# Supplementary figures and images for: Decline in severe diarrhea hospitalizations after the introduction of rotavirus vaccination in Ghana: a prevalence study
Source: BMC Infect Dis. 2014 Aug 6;14:431. doi: 10.1186/1471-2334-14-431 (PMC4132910; doi:10.1186/1471-2334-14-431)

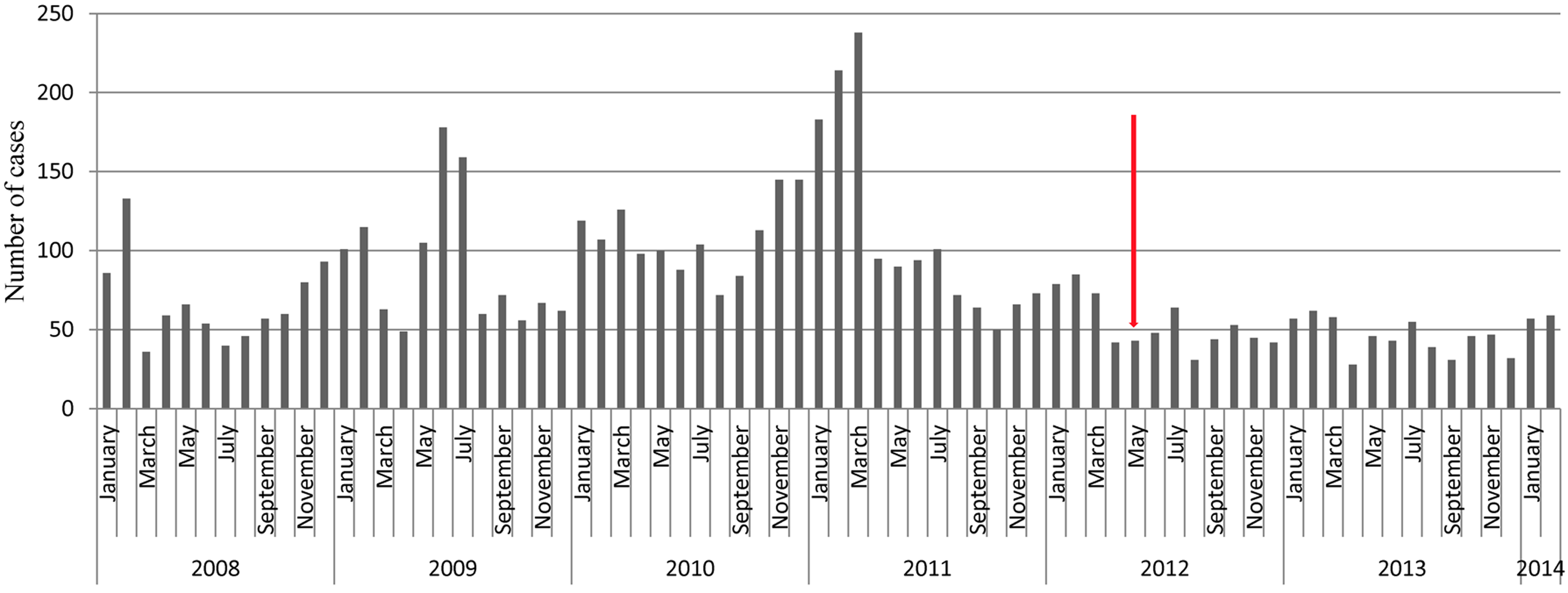

Supplement: Supplementary file 1 — Authors’ original file for figure 1 [file 12879_2014_3732_MOESM1_ESM.tif]

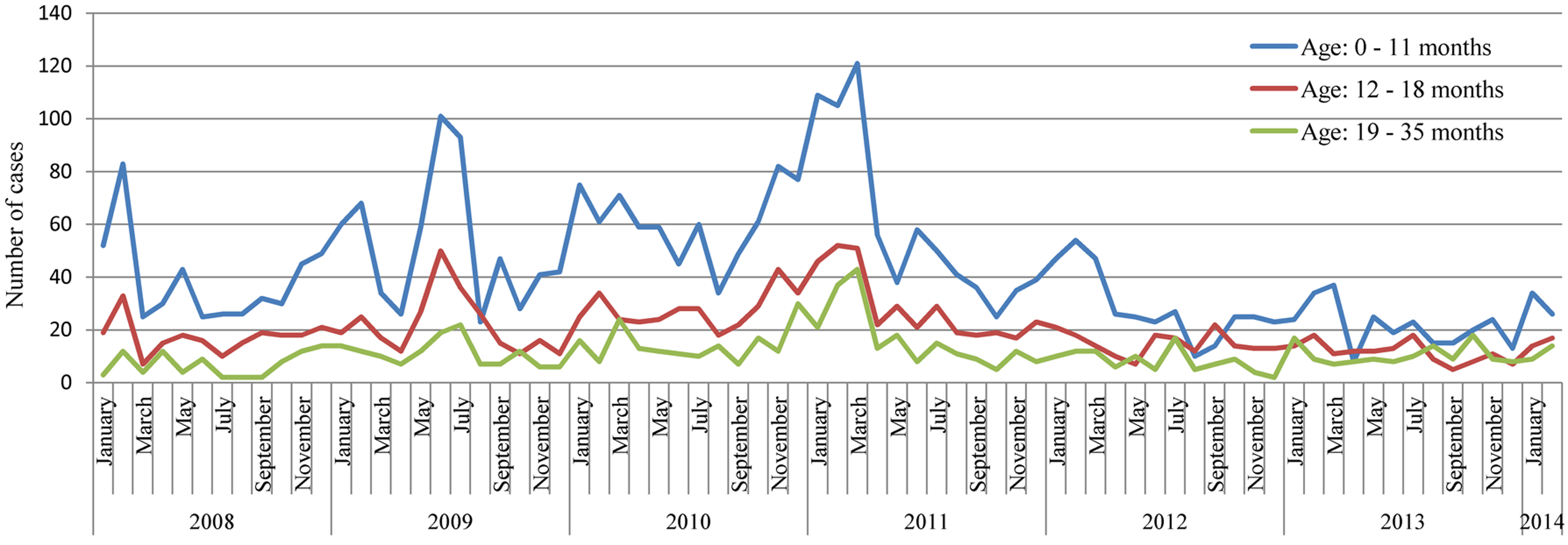

Supplement: Supplementary file 2 — Authors’ original file for figure 2 [file 12879_2014_3732_MOESM2_ESM.tif]

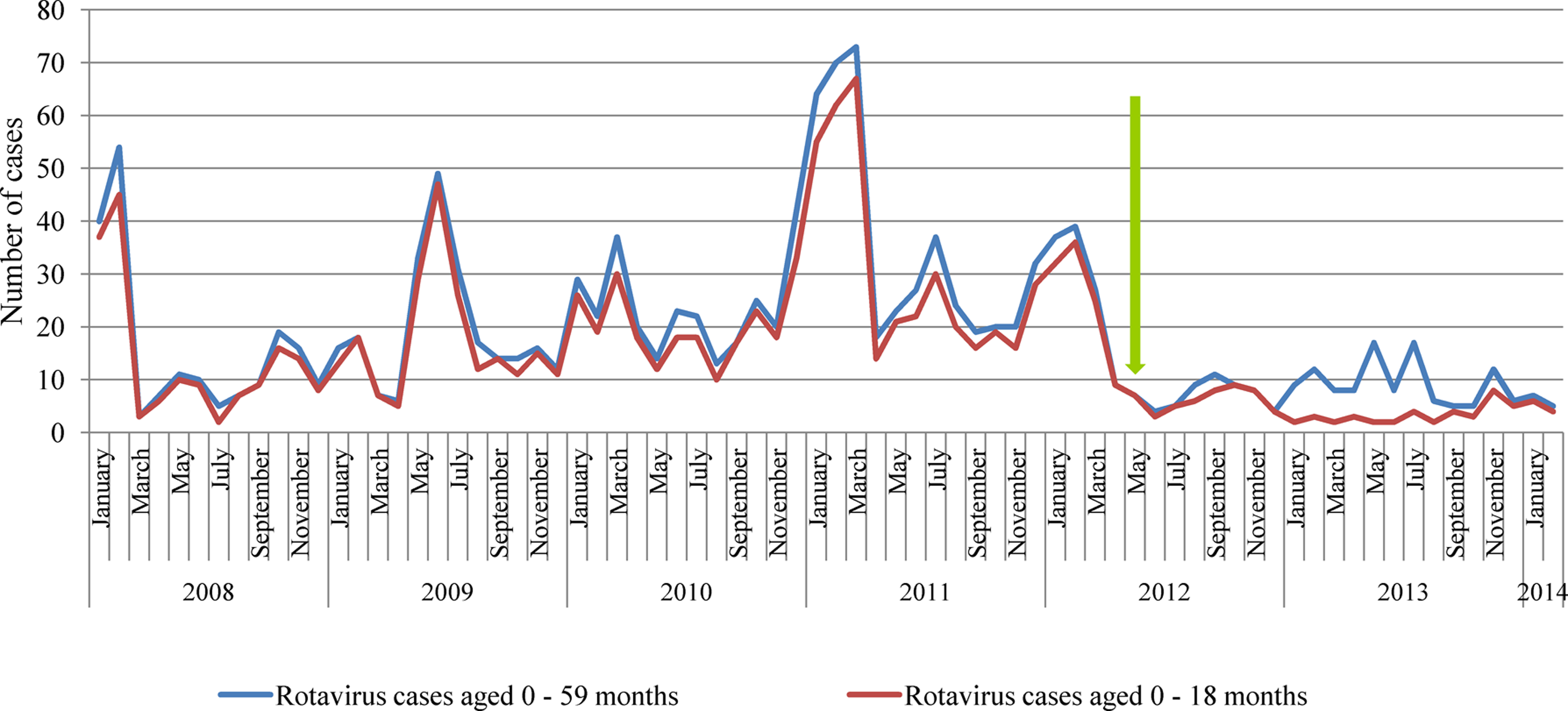

Supplement: Supplementary file 3 — Authors’ original file for figure 3 [file 12879_2014_3732_MOESM3_ESM.tif]
